# Efficacy of resin infiltration to mask post-orthodontic or non-post-orthodontic white spot lesions or fluorosis — a systematic review and meta-analysis

**DOI:** 10.1007/s00784-021-03931-7

**Published:** 2021-06-09

**Authors:** S. Bourouni, K. Dritsas, D. Kloukos, R. J. Wierichs

**Affiliations:** 1 Department of Restorative, Preventive and Pediatric Dentistry, zmk bern, University of Bern, Freiburgstrasse 7, 3010 Bern, Switzerland; 2 Department of Orthodontics and Dentofacial Orthopedics, zmk bern, University of Bern, Freiburgstrasse 7, 3010 Bern, Switzerland

**Keywords:** Resin infiltration, White spot lesions, Fluorosis, Post-orthodontic, Fluoride varnish, Review, White spots, Tooth sealants, Fixed orthodontic appliances, Enamel microabrasion, Meta-analysis

## Abstract

**Objective:**

The present review systematically analyzed clinical studies investigating the efficacy of resin infiltration on post-orthodontic or non-post-orthodontic, white spot lesions (WSL), or fluorosis.

**Materials:**

Five electronic databases (Central, PubMed, Ovid MEDLINE, Ovid EMBASE, LILACS) were screened. Article selection and data abstraction were done in duplicate. No language or time restrictions were applied. Outcomes were visual-tactile or DIAGNOdent measurements.

**Results:**

Eleven studies with 1834 teeth being affected in 413 patients were included. Nine studies were randomized control trials, one a prospective cohort study, and one had an unclear study design. Meta-analysis could be performed for “resin infiltration vs. untreated control,” “resin infiltration vs. fluoride varnish,” and “resin infiltration without bleaching vs. resin infiltration with bleaching.” WSL being treated with resin infiltration showed a significantly higher optical improvement than WSL without any treatment (standard mean difference (SMD) [95% CI] = 1.24 [0.59, 1.88], moderate level of evidence [visual-tactile assessment]) and with fluoride varnish application (mean difference (MD) [95% CI] = 4.76 [0.74, 8.78], moderate level of evidence [DIAGNOdent reading]). In patients with fluorosis, bleaching prior to resin infiltration showed no difference in the masking effect compared to infiltration alone (MD [95% CI] = − 0.30 [− 0.98, 0.39], moderate level of evidence).

**Conclusion:**

Resin infiltration has a significantly higher masking effect than natural remineralization or regular application of fluoride varnishes. However, although the evidence was graded as moderate, this conclusion is based on only very few well-conducted RCTs.

**Clinical relevance:**

Resin infiltration seems to be a viable option to esthetically mask enamel white spot lesions and fluorosis.

**Supplementary Information:**

The online version contains supplementary material available at 10.1007/s00784-021-03931-7.

## Introduction

Enamel opacities occur as a consequence of damage of the dental follicle during eruption, disturbances during enamel development or cariogenic activity in the case of improper oral hygiene [1]. The latter often associated with fixed orthodontic appliances since fixed elements represent an additional retention opportunity for biofilm and therefore increase the caries risk [2, 3]. Due to their whitish, opaque, and chalky appearance caused by mineral loss in enamel [4], these lesions are often termed white spot lesions (WSL). The appearance of WSL can be physically explained by the stronger scattering of light within the subsurface demineralized enamel as a result of air and saliva inclusions in comparison to the surrounding healthy enamel [5] and can persist for more than 10 years after removal of the orthodontic appliances [6], thus, being an esthetic burden for the patients [7], especially when anterior teeth are affected.

These white spot lesions remineralize once the brackets have been removed. Although fluoride-containing agents can be used to enhance remineralization, the esthetic appearance is usually not sufficiently improved [8]. Several preventive strategies have been employed to avoid the initiation, to arrest or reverse the progression, or to mask the WSL. During treatment with fixed elements, sealants or bonding agents — being applied before [9] or after [10] the fixed elements are bonded — as well as fluoride- or chlorhexidine-containing mouthwashes [11] as well as casein phosphor peptide amorphous calcium phosphate containing pastes (CPP-ACP) [12] have been proposed. Furthermore, after the removal of the fixed elements fluoride-containing agents [8], CPP-ACP-containing pastes [13] or bioactive glasses can be used to enhance remineralization [14] have been shown to enhance remineralization. However, all these strategies cannot (completely) prevent the development of white spot lesions [12, 15] or treatment options have not yet been tested under clinical situation. The esthetic appearance most often remains impaired [13, 16]. Microabrasion represents another treatment option which is most suitable for very superficial lesions. In the case of deeper lesions, concave tooth surfaces may result [17]. Direct and indirect restorations also lead to satisfactory and predictable results, but these should be used mainly in cavitated lesions [18].

By resin infiltration, the microporous enamel areas of non-cavitated initial carious lesions are obturated by low-viscosity light-cured resins (infiltrants) [19], thus, inhibiting further caries progression [19, 20]. Apart from caries inhibition, resin infiltration is also able to mask white spot lesions [21]. As the refractive index of the infiltrant (1.52) is close to the index of enamel/apatite (1.62), as opposed to the indices of water (1.33) and air (1.00), light scattering is reduced with increasing degree of infiltration [22, 23].

A recent systematic review not only highlighted that resin infiltration seems to be a feasible option for color masking of enamel whitish discolorations [24], but also highlighted the lack of (randomized controlled) trials and the inadequate follow-up periods to assess the long-term results of this technique. However, in the last 5 years, several new (randomized controlled) trials have been published. Therefore, the aim of this systematic review and meta-analysis was to critically summarize the literature and evaluate the long-term efficacy of resin infiltration therapy with regard to esthetic appearance and long-term stability of the results.

## Materials and methods

### Review design

No study registration is necessary for this review. This review was conducted and reported according to the PRISMA statement [25]. The PICOS model was used to define the in- and exclusion criteria and, thus, to structure the clinical research question [26] (Table 1). Thus, the present review aimed at systematically retrieving and analyzing clinical studies investigating the efficacy of resin infiltration to mask WSL or fluorosis.

**Table 1:** PICOS schema: population (P), intervention (I), comparison (C), outcomes (O), and study design (S)

P	-	Participants: patients of any age with WSL or fluorosis
I	-	Intervention: resin infiltration
C	-	Control: any other (placebo) treatment or untreated control
O	-	Outcome: primary: any esthetic outcome; secondary: patient-related outcome measures (PROMs) such as pain, satisfaction, discomfort, quality of life indicators, and economic factors
S	-	Studies: randomized controlled clinical trials (RCTs), prospective controlled clinical trials (CCTs), prospective and retrospective cohort studies, and studies with split-mouth and parallel-arm designs

### Search strategy

Detailed search strategies were developed and appropriately revised for each database, considering the differences in controlled vocabulary and syntax rules by two authors (S.B., K.D.) (the search strategies for Medline/PubMed are shown in Supplementary material Table 1). The following electronic databases were searched to find reports of relevant published studies:

- The Cochrane Central Register of Controlled Trials (CENTRAL) (up to December 31, 2019);
- MEDLINE (PubMed) (1946 to December 31, 2019);
- Ovid MEDLINE (In-Process & Other Non-Indexed Citations, December 31, 2019;
- Ovid EMBASE (1947 to December 31, 2019)
- LILACS (1982 to December 31, 2019)

Two authors (S.B., K.D.) independently reviewed title and abstract using these search strategies. The reviewers were not blinded to the identity of the journal names or article authors, their institutions, or the results of their research. No language or time restrictions were applied. A detailed sequence of filtering search results to include relevant articles can be found in the Supplementary material. In order to further identify potential articles for inclusion, grey literature was searched in the register of clinical studies hosted by the US National Institutes of Health (www.clinicaltrials.gov), the multidisciplinary European database (www.opengrey.eu), the National Research Register, and Pro-Quest Dissertation Abstracts and Thesis databases. Agreement concerning study inclusion or data extraction was achieved by consultation and discussion with a third author (D.K.). Selected articles were screened full-text. Cross-referencing was performed to identify further articles to be assessed.

### Data collection

Two authors performed data extraction independently and in duplicate (S. B., K. D.). The following data were collected in predefined excel sheets: author/title/year of study, study affiliation data, study type and setting, design of the study, number/age/gender of patients, intervention applied, inclusion criteria and outcome definitions, outcome assessed with all relevant clinical variables (visual-tactile, laser fluorescence, colorimetric analysis, overall lesion size), drop-outs, follow-up (maximum follow-up over all groups was used), sources of funding, trial registration, and publishing of the trial’s protocol.

For longitudinal studies and clinical trials presented in different journals or in different publication years, results were presented within one column.

In case of missing data, it was attempted to contact the corresponding author via e-mail. Studies without enough data for meta-analyses were kept in the systematic review but excluded from the meta-analyses.

### Data synthesis and grading

Meta-analyses were to be conducted if studies with similar comparisons reported the same outcomes. For continuous variables, the primary measures of effect between treatment and control groups were the mean differences (MD) for studies using the same outcome and standardized mean differences (SMD) for studies using the same construct but different scales.

Statistical heterogeneity was assessed using a chi^2^ test and the *I*^2^ statistic [27]. Fixed or random-effects meta-analysis was performed depending on heterogeneity (*I*^2^ < 35%: fixed-effects; *I*^2^ > 35%: random-effect) [28]. Risk of bias for interventional, randomized controlled trials (RCTs) was performed using the Risk of Bias 2.0. tool [29] and for interventional, non-randomized controlled trials using the ROBINS-I tool [30]. Grading of evidence was performed according to the GRADE network levels using Grade Profiler 3.6 [31]. Publication bias was assessed by Funnel plots [32].

To avoid unit-of-analysis errors, the guidelines outlined by the Cochrane collaboration (chapter 9.3.4.) were followed [33]. Therefore, baseline data were compared with data of a single time point (mostly longest follow-up period).

## Results

**Fig. 1:**
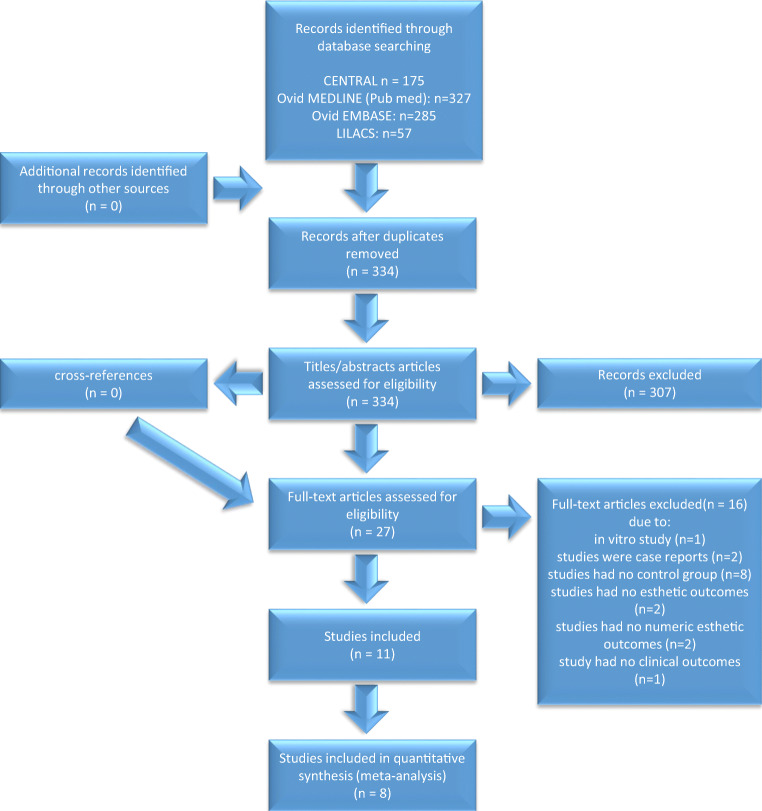
Study flow

A total of 334 studies were initially identified, and after title and abstract screening, 28 studies were assessed for eligibility. After full-text screening, 16 studies were excluded (Fig. 1, Supplementary material Table 2). Eventually, 11 studies with 1834 teeth being affected in 413 patients, 8–30 years of age, were included. Nine studies were randomized control trials [34–42], one a prospective cohort study [43], and one did not report if it was a randomized or non-randomized study [44], all of which investigated the efficacy of resin infiltration on post-orthodontic [34, 37, 39, 41–43] or non-post-orthodontic [35, 36] white spot lesions or fluorosis [38, 40, 44]. Resin infiltration was compared to fluoride varnish [35, 36, 42, 43], untreated control [34, 36, 39, 41], microabrasion [37, 44], or bleaching [38, 40]. The outcomes were described using DIAGNOdent values [35, 36, 42, 43], spectroscopy [37, 39, 42, 44], colorimetric values [40], ICDAS II [41, 43] /LAA-ICDAS scores [36], or a modified enamel decalcification score [34]. Furthermore, visual analog scales (VAS) were used to evaluate the change/improvement in esthetics [38, 41] or patients’ oral health-related quality of life [40]. An overview of the main characteristics of the included studies is presented in the Supplementary material Table 3.

Meta-analyses were performed for studies with similar interventions and outcome measures investigated in more than one study. Although analysis showed that a meta-analysis could be performed for resin infiltration vs. untreated control [34, 36, 39, 41] and for resin infiltration vs. fluoride varnish [35, 36, 42, 43], one study in each comparison had to be excluded since not all information required for recalculation was reported [36] or the inclusion criteria of the lesions were inconsistent [42]. Even if not differentiating between untreated controls and fluoride varnish controls, no further study could have been included in meta-analysis. Either data were not reported for recalculation (e.g., color difference (ΔE values) [42]) or data were presented insufficiently — e.g., reporting an ordinally scaled outcome (ICDAS II score) by using values for continuous outcome [43].

**Fig. 2:**
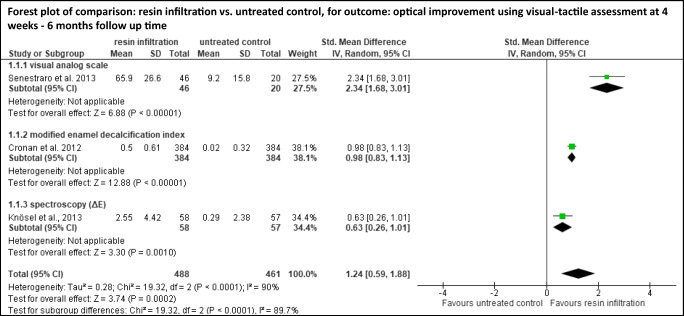
Quantitative meta-analyses for the comparison resin infiltration vs. untreated control. Standardized mean differences (SMD) (and 95% confidence intervals (95% CI)) were calculated since studies used the same construct but different scales. Forest plots, heterogeneity parameter (*I*^2^), as well as overall statistics (*Z*, *P*) are given

**Fig. 3:**
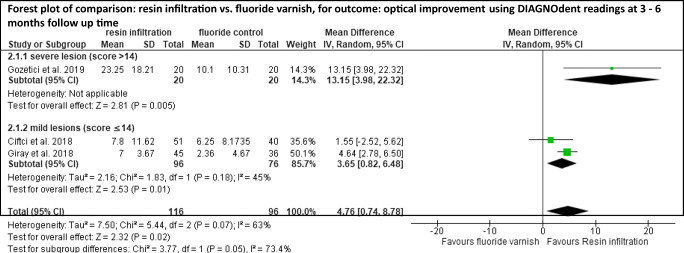
Quantitative meta-analyses for the comparison resin infiltration vs. fluoride control. Mean differences (SMD) (and 95% confidence intervals (95%CI)) were calculated since studies used the same construct and same scales. Forest plots, heterogeneity parameter (*I*^2^), as well as overall statistics (*Z*, *P*) are given

WSL being treated with resin infiltration showed a significantly higher optical improvement than WSL being remineralized by saliva without any additional treatment (SMD [95% CI] = 1.24 [0.59, 1.88], visual-tactile assessment) [34, 39, 41] (Fig. 2). Furthermore, WSL being treated with resin infiltration showed a significantly higher optical improvement than WSL being treated with fluoride varnish (MD [95% CI] = 4.76 [0.74, 8.78], DIAGNOdent readings) [35, 36, 43] (Fig. 3). However, baseline values and cut-off points for DIAGNOdent classification within the comparison as well as within each study varied widely.

Three studies investigated the masking effect of resin infiltration in patients with fluorosis [38, 40, 44]. Here, resin infiltration alone was compared to either (1) bleaching plus resin infiltration [38, 40], (2) bleaching without any further treatment [38], or to microabrasion [44]. When teeth were bleached a few days before they were infiltrated, no improvement in the masking effect could be observed compared to infiltration alone (SMD [95% CI] = − 1.53 [− 4.75, 1.70], patient/dentist satisfaction) [38, 40] (Supplementary material Figure 1), whereas a significantly higher masking effect was observed for resin infiltration alone compared with bleaching alone (MD [95% CI] = 3.97 [3.33, 4.61], dentist satisfaction) [38]. However, the effect of bleaching before infiltration was not analyzed and, thus, remains unclear.

### Quality assessment

**Fig. 4:**
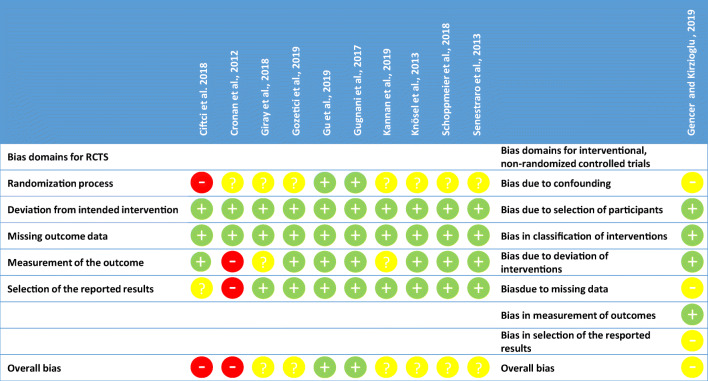
Risk of bias assessment. **a** For interventional, randomized controlled trials (RCTs) Risk of Bias 2.0 tool and **b** for interventional, non-randomized controlled trials the ROBINS-I tool was used

Of the 11 trials, quality of 2 was assessed as low [37, 38], of 7 rated with concerns [35, 36, 40–42, 44, 45] and of another 2 as high risk of bias [34, 43] (Fig. 4). Most of the studies did not report adequately the randomization process or any methods of allocation concealment. However, a blinded outcome assessor was recruited in most of the studies, where blinding of the doctors or the patients was not feasible.

Grading of evidence for meta-analyses showed moderate level of evidence for resin infiltration compared to untreated controls or to fluoride varnish and for resin infiltration with or without prior bleaching (Supplementary material Table 4).

## Discussion

In this systematic review, the effect of resin infiltration on white spot lesions and fluorosis has been critically summarized. A wide variety of studies, in which resin infiltration was compared to fluoride varnish, untreated control, microabrasion, or bleaching, has been extracted. The outcomes were described using DIAGNOdent, spectroscopy, photographic images, as well as ICDAS II/LAA-ICDAS scores or a modified enamel decalcification score. The median follow-up period was only 6 months with a range between 1 day [38] and 12 months [37]. This reflects that obviously no “gold standard” protocol to analyze masking effects has been agreed yet. However, the present meta-analysis also showed that resin infiltration has a significantly higher masking effect than natural remineralization or regular application of fluoride varnishes.

Regardless of the used outcome, resin infiltration showed a significantly higher optical improvement of WSL than remineralization alone (untreated WSL) as well as significantly higher optical improvement than the regular application of fluoride varnishes. Only in one study, fluoride varnish provided optical results comparable to resin infiltration [42]. However, in this study, inconsistencies of the inclusion criteria of the lesions were reported. Furthermore, after fluoride application, the optical improvement required up to 6 months, whereas after resin infiltration, a subsequent improvement could be observed.

In the included studies, the follow-up periods ranged between 1 day and 12 months with a median follow-up time of 6 months. Although it can be expected that natural remineralization from saliva can be enhanced with a study design which lasts at least 21 days [46, 47] — which was the case for all studies except one [38] — even a 6 months follow-up period seems to be rather short when compared to the advised 3 year follow-up for direct restauration and the advised 5 year follow-up for indirect restauration [48]. However, the short follow-up periods may be explained by two facts: (1) the use of resin infiltration to mask WSL is a relatively new technique, and (2) in case of a masking effect in the test group (resin infiltration) and no masking effect in the (untreated or treated) control group, it might be necessary to also infiltrate the control teeth after a predefined observation period to obtain an ethical approval [39, 49]. In one of the mentioned studies [39], WSL being infiltrated were followed up to 3.8 years. After a mean (non-controlled) follow-up period of 2.8 years, a significant masking effect could still be observed when compared to the situation before infiltration, but no significant difference could be observed between 0.5 and 2.8 years after infiltration. Nonetheless, studies with long-term follow-ups are still required to confirm the stability of the esthetic results.

The results of the present meta-analyses are also consistent with several non-controlled trials [21, 39, 50, 51]. After observation periods of 1 week and 12 months, more than 351 infiltrated post-orthodontic WSL in more than 79 patients were significantly masked. However, only a few studies reported if the significant improvement in the visual appearance of the WSL was subjectively satisfying [21, 49, 50] or if (more objective) a reduction of the colorimetric value ΔΕ below 3.7 — the threshold for perception from a common social distance [52] — could be achieved [21, 37]. In the first study, for at least 98 teeth being etched once or twice the results were satisfying, whereas no information was given on the 123 teeth being etched three times [21]. However, in this group, 37% of the lesions showed a reduction of ΔΕ below 3.7. In the second study, patients receiving infiltration were the most satisfied patients compared to the patients in both control groups (natural remineralization and professional fluoride application) [49]. However, no numeric results were presented. In the third study, 61% of the lesions were completely masked and 33% partially [50], and in the fourth study, a reduction of the ΔΕ-values below 3.7 was reported [37].

For post-orthodontic, WSL lesions were infiltrated after bracket removal. However, not all studies reported the time between bracket removal and infiltration [21, 34, 37, 41, 42, 45, 49, 50] and only in a few studies, teeth were infiltrated within 1 year after brackets removal [21, 42, 45, 49]. One of these studies reported that the time interval between bracket removal and infiltration seems to play an important role to effectively mask white spot lesions [45]. The shorter this time interval, the more successful the masking effect appears to be. Consequently, the question as how the masking effect could be further improved by infiltration during orthodontic treatment was raised. Until now, this question has only be investigated in one case report [53] (being presented by the manufacturer of Icon) and one non-controlled trial [54]. In both publications, after diagnosing WSL during orthodontic treatment, WSL were infiltrated without interrupting the orthodontic treatment. However, this approach has not yet been further analyzed although the results were promising.

Colorimetric analysis with imaging software and spectrophotometry or digital photographic cameras was widely used for outcome assessment. Using these methods, each color is described in the CIE L*a*b* color system: It records colorimetric parameters three-dimensionally: lightness (L*), green-red chromaticity (a*), and blue-yellow chromaticity (b*) [22], and the color difference ΔΕ is defined by the CIE76 formula [22]: the square root of the sum of the squared differences of each of the three color values between two different points. Consequently, a perfect color match would give the value of 0. However, it has to be noted that values obtained with the use of a photographic camera can differ to these obtained with a spectrophotometer [55]. This can presumably also be seen in the present review. After resin infiltration, an increase of the L*-value was observed for some studies using a spectrophotometer [42, 44], whereas a decrease of the L*-value was observed for studies using a photographic camera [21, 40] and for some studies using a spectrophotometer [45]. The different behavior of the L*-value can be attributed to the fact that spectrophotometer measures light absorption and that L^*^-values increase as less light gets scattered within the lesion, after the infiltrant is applied. Contrastingly, on digital photographs, L*-values decrease as the lesion appears less white after treatment. Consequently, when comparing colorimetric values, the measures used to calculate the values should be kept in mind, especially when absolute values are compared.

DIAGNOdent has been accepted for its reproducibility and sensitivity over conventional radiography in primary occlusal caries in vitro [56, 57] and in vivo [58]. It has also been evaluated for secondary caries adjacent to amalgam restorations or composite restorations [59]. However, contradictory results on the evaluation for DIAGNOdent readings under restorative materials have been reported [60, 61]. Although DIAGNOdent readings seem to show the same sensitivity and specificity as digital radiographs [61], the reading can be affected by dental materials [60]. In respect to low-viscosity resins (e.g., resin infiltration), it has not been analyzed yet if DIAGNOdent readings are affected by the low-viscosity resin and if there is a correlation between the DIAGNOdent value and optical outcomes (e.g., ΔE-values). Only one in vitro study analyzed if the DIAGNOdent values correlate with subjective visual assessments and laser fluorescence readings [62]. Although the esthetic appearance of the artificial lesions was significantly improved according to the visual assessment, no difference in the DIAGNOdent values was recorded between before and after infiltration. This is also in agreement with unpublished data of the authors: No correlation between the change of DIAGNOdent values and colorimetric values (ΔE) or the subjective assessment could be observed when investigating infiltration. Consequently, even if several studies used DIAGNOdent measurements to measure the masking abilities [35, 36, 42, 43, 49], the results of the respective studies have to be interpreted with caution.

In the included studies, infiltration was performed according to the manufacturer’s recommendation. Even the number of etching steps varied within the manufacturer’s specification (maximum number of 3 procedures). Only in one study, 55 teeth (out of 111) were etched more than three times [45]. Nonetheless, in most of the studies, the number of etching procedures was predefined by the study protocol [34–36, 40–44], and only in a few studies, the number of etching procedures was individualized by using the re-wetting process [37, 38, 45]. Thus, it might be speculated that the significant masking effect being observed in the present meta-analysis could have been further improved, if a more individualized etching protocol had been used in all studies.

## Conclusion

Resin infiltration has a significantly higher masking effect than natural remineralization or regular application of fluoride varnishes. Thus, it seems to be a viable option to esthetically mask enamel white spot lesions and mild to medium fluorosis. However, although the evidence was graded as moderate, this conclusion is based on only few well-conducted RCTs with moderate to high risks of bias.

## Supplementary Information

ESM 1 (DOCX 46 kb)

ESM 2 (DOCX 33 kb)

ESM 3 (PPTX 53 kb)
