# Supplementary material for: Efficacy of resin infiltration to mask post-orthodontic or non-post-orthodontic white spot lesions or fluorosis — a systematic review and meta-analysis
Source: Clin Oral Investig. 2021 Jun 9;25(8):4711–9. doi: 10.1007/s00784-021-03931-7 (PMC8342329; doi:10.1007/s00784-021-03931-7)
Supplement: Supplementary file 1 — (DOCX 46 kb) [file 784_2021_3931_MOESM1_ESM.docx]

**Efficacy of resin infiltration to mask post-orthodontic or non-post orthodontic white spot lesions or fluorosis - A systematic review and meta-analysis**

**Supplementary material**

Sequence of filtering search results

The sequence of filtering search results in order to include relevant articles in the review was as follows:

1. articles written in German, Greek or English,
2. articles written in languages of which the authors were able to get a vague idea of the content (Latin-based languages) or those having only a translated (English, Greek or German) abstract,
3. articles written in languages the authors were not able to understand at all.

After deciding on the German, Greek and English articles the authors read through the category 2) articles realizing that only one of them fulfilled inclusion criteria. Of course, for all category 3) articles (three studies), the authors were not able to make a decision based on their content.

**Supplementary material figure 1:**

Quantitative meta-analyses for the comparison resin infiltration vs. bleaching plus subsequent resin infiltration. Standardized mean differences (SMD) (and 95% confidence intervals (95%CI)) were calculated since studies used the same construct but different scales. Forest plots, heterogeneity parameter (I2) as well as overall statistics (Z, P) are given.

**Supplementary table 1:** Search strategy as used for Pubmed

| Search | Query | Results |
| --- | --- | --- |
| #1 | resin infiltration | 753 |
| #2 | resin infiltration[tiab] | 315 |
| #3 | (resin infiltration[tiab]) AND (enamel[Title/Abstract] OR defect*[Title/Abstract]) | 148 |
| #4 | (resin infiltration[tiab]) AND white spot lesion[Title/Abstract] | 18 |
| #5 | (resin infiltration[tiab]) AND orthodont*[Title/Abstract] | 29 |
| #6 | (resin infiltration[tiab]) AND (defect*[Title/Abstract] OR hypoplas*[Title/Abstract] OR discolor*[Title/Abstract]) | 27 |
| #7 | (acrylic resins[MeSH Terms]) AND infiltration[Title/Abstract] | 222 |
| #8 | ((resin infiltration[tiab]) AND enamel[Title/Abstract]) AND (defect*[Title/Abstract] OR hypoplas*[Title/Abstract] OR amelogenesis[Title/Abstract] OR stain[Title/Abstract] OR fluorosis[Title/Abstract]) | 22 |
| #9 | dental fluorosis[MeSH Terms] | 2518 |
| #10 | (dental fluorosis[MeSH Terms]) AND infiltration[Title/Abstract] | 14 |
| #11 | (amelogenesis imperfecta[MeSH Terms]) AND resin infiltration[Title/Abstract] | 1 |
| #12 | (tooth demineralization[MeSH Terms]) AND resin infiltration[Title/Abstract] | 123 |
| #13 | (dental enamel[MeSH Terms]) AND infiltration[Title/Abstract] | 175 |
| #14 | (calcification, tooth[MeSH Terms]) AND infiltration[Title/Abstract] | 1 |
| #15 | (tooth discoloration[MeSH Terms]) AND infiltration[Title/Abstract] | 19 |
| #16 | (synthetic resin[MeSH Terms]) AND infiltration[Title/Abstract] | 646 |
| #17 | (((synthetic resin[MeSH Terms]) AND infiltration[Title/Abstract])) AND (enamel[Title/Abstract] OR orthodont*[Title/Abstract]) | 170 |
| #18 | (dental white spots[MeSH Terms]) AND infiltration[Title/Abstract] | 189 |

The results of all search queries (except #1, #9 and #16) have been imported as bibliographies in Zotero (Coporation for Digital Scholarship, Vienna, Virginia, USA). Then the results have been combined and duplicates have been excluded.

**Supplementary table 2**: Excluded studies

| Author (Year) | Reason for exclusion |
| --- | --- |
| (1) | No control group |
| (2) | No esthetic outcomes reported |
| (3) | No control group |
| (4) | No control group |
| (5) | Both groups were intervention groups without control |
| (6) | No numeric esthetic outcomes reported |
| (7) | No numeric esthetic outcomes reported |
| (8) | No control group |
| (9) | No control group |
| (10) | No control group |
| (11) | Case report |
| (12) | No control group |
| (13) | No esthetic outcomes reported |
| (14) | Case report |
| (15) | In vitro study |
| (16) | Missing clinical outcomes |

**Supplementary material table 4**: Quality assessment

**WSL**

| **Quality assessment** | | | | | | | **No of patients** | | **Effect** | | **Quality** | **Importance** |
| --- | --- | --- | --- | --- | --- | --- | --- | --- | --- | --- | --- | --- |
|  |  |  |  |  |  |  |  |  |  |  |  |  |
| **No of studies** | **Design** | **Risk of bias** | **Inconsistency** | **Indirectness** | **Imprecision** | **Other considerations** | **RI** | **Untreated control** | **Relative (95% CI)** | **Absolute** |  |  |
| **Resin infiltration vs. untreated control (follow-up median 8 months; measured with: clinical variables; Better indicated by lower values)** | | | | | | | | | | | | |
| 3 | randomised trials | serious^1^ | no serious inconsistency | no serious indirectness | no serious imprecision | none | 488 | 461 | - | SMD 1.24 higher (0.59 to 1.88 higher) | ⊕⊕⊕O MODERATE |  |
| **resin infiltration vs. fluoride control (follow-up mean 5 months; measured with: laser flouroscence; Better indicated by lower values)** | | | | | | | | | | | | |
| 3 | randomised trials | serious^2^ | no serious inconsistency^1^ | no serious indirectness | no serious imprecision | none | 116 | 96 | - | MD 4.76 higher (0.74 to 8.78 higher) | ⊕⊕⊕O MODERATE |  |

^1^ see bias grading

**Fluorosis**

| **Quality assessment** | | | | | | | **No of patients** | | **Effect** | | **Quality** | **Importance** |
| --- | --- | --- | --- | --- | --- | --- | --- | --- | --- | --- | --- | --- |
|  |  |  |  |  |  |  |  |  |  |  |  |  |
| **No of studies** | **Design** | **Risk of bias** | **Inconsistency** | **Indirectness** | **Imprecision** | **Other considerations** |  | **Control** | **Relative (95% CI)** | **Absolute** |  |  |
| **resin infiltration vs. bleaching + resin infiltration (follow-up 1-180 days; measured with: optical improvement; Better indicated by lower values)** | | | | | | | | | | | | |
| 2 | randomised trials | no serious risk of bias | no serious inconsistency | no serious indirectness | serious^1^ | none | 212 | 233 | - | SMD 1.53 lower (4.75 lower to 1.7 higher) | ⊕⊕⊕O MODERATE |  |

^1^ 95% confidence interval includes no effect and the upper or lower confidence limit crosses the minimal important difference (MID).

**References**

1. Bhandari R, Thakur S, Singhal P, Chauhan D, Jayam C, Jain T. Concealment Effect of Resin Infiltration on Incisor of Grade I Molar Incisor Hypomineralization Patients: An in Vivo Study. *J Conserv Dent*. 2018;**21**(4):450-54.

2. Caglar E, Kuscu OO, Hysi D. Four Year Evaluation of Proximal Resin Infiltration in Adolescents. *Acta Stomatol Croat*. 2015;**49**(4):304-8.

3. Feng C, Liu R, Liu R, Zhao Q, Chu X. [Effect of Infiltration Resin on the Color Masking of Labial Enamel White Spot Lesions]. *Hua Xi Kou Qiang Yi Xue Za Zhi*. 2013;**31**(6):597-9.

4. Feng CH, Chu XY. [Efficacy of One Year Treatment of Icon Infiltration Resin on Post-Orthodontic White Spots]. *Beijing Da Xue Xue Bao Yi Xue Ban*. 2013;**45**(1):40-3.

5. Hammad SM, El Banna M, El Zayat I, Mohsen MA. Effect of Resin Infiltration on White Spot Lesions after Debonding Orthodontic Brackets. *Am J Dent*. 2012;**25**(1):3-8.

6. Hasmun N, Lawson J, Vettore MV, Elcock C, Zaitoun H, Rodd H. Change in Oral Health-Related Quality of Life Following Minimally Invasive Aesthetic Treatment for Children with Molar Incisor Hypomineralisation: A Prospective Study. *Dent J (Basel)*. 2018;**6**(4).

7. Jumanca D, Atena G, Podariu A, Ardelean L, Rusu L. Infiltration Therapy - an Alternative to Fluoride Varnish Application for Treatment of White Spot Lesion after Fixed Orthodontic Treatment. *Revista de Chimie*. 2012;**63**:783-86.

8. Kim S, Kim EY, Jeong TS, Kim JW. The Evaluation of Resin Infiltration for Masking Labial Enamel White Spot Lesions. *Int J Paediatr Dent*. 2011;**21**(4):241-8.

9. Kobbe C, Fritz U, Wierichs RJ, Meyer-Lueckel H. Evaluation of the Value of Re-Wetting Prior to Resin Infiltration of Post-Orthodontic Caries Lesions. *J Dent*. 2019;**91**:103243.

10. Mazur M, Westland S, Guerra F, Corridore D, Vichi M, Maruotti A, et al. Objective and Subjective Aesthetic Performance of Icon(R) Treatment for Enamel Hypomineralization Lesions in Young Adolescents: A Retrospective Single Center Study. *J Dent*. 2018;**68**:104-08.

11. Muthuvel P, Ganapathy A, Subramaniam MK, Revankar VD. Erosion Infiltration Technique': A Novel Alternative for Masking Enamel White Spot Lesion. *J Pharm Bioallied Sci*. 2017;**9**(Suppl 1):S289-s91.

12. Ogodescu A, Ogodescu E, Talpos S, Zetu I. [Resin Infiltration of White Spot Lesions During the Fixed Orthodontic Appliance Therapy]. *Rev Med Chir Soc Med Nat Iasi*. 2011;**115**(4):1251-7.

13. Peters MC, Hopkins AR, Jr., Yu Q. Resin Infiltration: An Effective Adjunct Strategy for Managing High Caries Risk-a within-Person Randomized Controlled Clinical Trial. *J Dent*. 2018;**79**:24-30.

14. Román-Rodríguez JL, Agustín-Panadero R, Roig-Vanaclocha A, Amengual J. A Tooth Whitening and Chemical Abrasive Protocol for the Treatment of Developmental Enamel Defects. *J Prosthet Dent*. 2020;**123**(3):379-83.

15. Silva LO, Signori C, Peixoto AC, Cenci MS, Faria ESAL. Color Restoration and Stability in Two Treatments for White Spot Lesions. *Int J Esthet Dent*. 2018;**13**(3):394-403.

16. Wang L, Jian J, Lu HF. Efficiency of Resin Infiltration Versus Fluride Varnish for Treatment of Post-Orthodontic White Spot Lesions. *Chinese Journal of Tissue Engineering Research*. 2013;**17**:5303-08.
