# Supplementary material for: Efficacy of resin infiltration to mask post-orthodontic or non-post-orthodontic white spot lesions or fluorosis — a systematic review and meta-analysis
Source: Clin Oral Investig. 2021 Jun 9;25(8):4711–9. doi: 10.1007/s00784-021-03931-7 (PMC8342329; doi:10.1007/s00784-021-03931-7)
Supplement: Supplementary file 2 — (DOCX 33 kb) [file 784_2021_3931_MOESM2_ESM.docx]

**Supplementary material table 3 (part 1)**: Detailed summary of included studies

| **First author** | **Type of lesion and tooth** | **time between debonding/first diagnose and treatment** | **Type of Intervention(s)** | **resin infiltration (frequency of etching)** | **Mean age of patients [range, mean (SD)]** | **Study design** | **Follow-up time(s)** | **Number of patients (M, F) at the beginning, number of teeth (per group)** | **Number of patients (M, F) at the end,  number of teeth (per group)** |
| --- | --- | --- | --- | --- | --- | --- | --- | --- | --- |
| **Ciftci et al. 2018** | post-orthodontic  WSL | 'recently' | **Hyopmineralization** - resin infiltration (RI) + OHI  (oral hygiene instruction) **WSL** - resin infiltration (RI) + OHI  - fluoride varnish (FV) + OHI | according to the manufacturer’s instructions (1x) | 8-16 yrs. | Prospective cohort | T0: Baseline T1d: after resin infiltration T1: 1 months T3: 3 months | 68 (41F, 27M) 137 teeth (41, 51, n/a) | 66 (41F, 25M) 132 teeth (41, 51, 40) |
| **Cronan et al. 2012** | post-orthodontic  WSL | 0-14 yrs. 7 within 0-6 months 4 within 7-14 yrs. | - resin infiltration (RI) - untreated control (NC) | according to the manufacturer’s instructions (2x) | 16.5 yrs. IQR: 10.4 | RCT Split-mouth | T0: Baseline T1d: after resin infiltration T6w: 4-6 weeks | 11 (7M, 4F) 184 teeth (499 lesions for both groups) | (384, 384) |
| **Gencer & Kırzıoglu 2019** | 1. Fluorosis  2. Incisor hypomineralization | n/a n/a | - resin infiltration (RI) - microabrasion + fluoride varnish (+CPP-ACP) (MA+CPP-ACP) - microabrasion + fluoride varnish (+ tricalcium phosphate) (MA+TCP) | according to the manufacturer’s instructions (3x) | 8-17 yrs. | n/a  presumably prospective parallel or split-mouth | T0: Baseline T1d: after resin infiltration T3: 3 months T6: 6 months | 100 (?M, ?F) 289 teeth   Fluorosis 128 (n/a) Hypomin. 161 (n/a) | patients n/a 251 teeth  Fluorosis 103 (38, 34, 31) Hypomin. 148 (49, 49, 50) |
| **Giray et al. 2018** | non-post-orthodontic  WSL upper incisors | n/a | - resin infiltration (RI) - fluoride varnish (FV) | according to the manufacturer’s instructions (1x) | 8-14 yrs. 10.8 ± 2.1 yrs. | RCT Parallel groups | T1d: after resin infiltration (only for RI) T6: 6 months | 23 (13M, 10F) 81 teeth (45, 36) | 23 (13M, 10F) 81 teeth (45, 36) |
| **Gözetici et al. 2018** | non-post-orthodontic WSL | n/a | - resin infiltration (RI) - P11-4 peptide - fluoride varnish (FV)  - untreated control (NC) | according to the manufacturer’s instructions (1x) | 12-21 yrs. 15.4 ± 2.5 yrs. | RCT Split-mouth | T0: Baseline  T1w: 1 week (only DD) T3: 3 months T6: 6 months | 21 (10M, 11F) 84 teeth (21, 21, 21, 21) (4 teeth per patient, 1 in each group) | 20 80 teeth (20, 20, 20, 20) (4 teeth per patient, 1 in each group) |
| **Gu et al. 2019** | post-orthodontic WSL anterior teeth | >3 months | - resin infiltration (RI) - microabrasion (MA) | according to the manufacturer’s instructions (1x - 3x) | 12-19 yrs. 16 yrs. | RCT Split-mouth | T0: Baseline T1w: 1 week T6: 6 months T12: 12 months | 20 (8M, 12F) 128 teeth (64, 64) | 16 (7M, 9F) 108 teeth (54, 54) |
| **Gugnani et al. 2017** | Fluorosis anterior teeth | n/a | - resin infiltration (RI) - in-office bleaching (standard control) - resin infiltration (RI) (prolonged application time) - in-office bleaching + resin infiltration (RI) (20 days later) | according to the manufacturer’s instructions (1x - 3x) | 6-12 yrs. | RCT | T0: Baseline T1d: after resin infiltration | 80  80 teeth (20, 20, 20, 20) | 80  80 teeth (20, 20, 20, 20) |
| **Kannan & Padmanabhan 2019** | post-orthodontic WSL | < 1 month (?) | - resin infiltration (RI)  - fluoride varnish (+ tricalcium phosphate) (FV) | according to the manufacturer’s instructions (1x) | 14-30 yrs. F: 18 ± 0.2 yrs.  M: 20 ± 0.5 yrs. | RCT Parallel groups | T0: Baseline  T1d. after resin infiltration  T3: 3 months T6: 6 months | 12 (5M, 7F)  240 lesions (124, 116) 240 lesions on 193 teeth | 11  233 lesions (124, 109) 233 lesions on 186 teeth |

| **First author** | **Type of lesion and tooth** | **time between debonding/first diagnose and treatment** | **Type of Intervention(s)** | **resin infiltration (frequency of etching)** | **Mean age of patients [range, mean (SD)]** | **Study design** | **Follow-up time(s)** | **Number of patients (M, F) at the beginning, number of teeth (per group)** | **Number of patients (M, F) at the end,  number of teeth (per group)** |
| --- | --- | --- | --- | --- | --- | --- | --- | --- | --- |
| **Knösel et al. 2013,2018,2019** | post-orthodontic WSL anterior teeth | 1-12 months | - resin infiltration (RI) - untreated control (NC) | manufacturer’s instructions were modified modified etching procedure (1x - 4x) | 12-19 yrs. 15.5 yrs. | RCT Split-mouth  (after 6 month all lesions were infiltrated) | T0: Baseline T1d: after resin infiltration T1w: 1 week T4w: 4 weeks T3: 3 months T6: 6 months T12: 12 months T24: 24 months | At. 6 months 21 135 teeth (67,68)  At 24 months 8 40 teeth | At 6 months: 20 115 teeth (58,57)  At 24 months:  8 40 teeth |
| **Schoppmeier et al. 2018** | Fluorosis | n/a | - in-office bleaching + resin infiltration (BI+RI) (14 days later) - placebo in-office bleaching + resin infiltration (RI) (14 days later) | according to the manufacturer’s instructions (3x) | n/a 24.8 ± 3.7 yrs. | RCT | T0: Baseline T1d: after (placebo) bleaching T2w: after resin infiltration T1. 1 months T3. 3 months T6. 6 months | 27  410 teeth (218, 192) | 26 405 teeth (213, 192) |
| **Senestraro et al. 2013** | post-orthodontic WSL  anterior teeth | >3 month up to 4 years | - microabrasion + subsequent resin infiltration (RI) - untreated control (NC) | according to the manufacturer’s instructions (2x) | 14-21 yrs. 16.6 ± 1.8 yrs. | RCT Parallel groups | T0: Baseline T1d: after infiltration T8w: 8 weeks | 30 66 teeth (46, 20) | 20 66 teeth (46, 20) |

**Supplementary material table 3 (part 2)**: Detailed summary of included studies

| **First author** | **Measure of outcome** | **outcome:**  **visual-tactile assessment** | **outcome: colorimetric analysis between the treated and sound enamel** | **outcome:**  **DIAGNOdent** | **outcome:  demineralized area reduction** | **narrative results** | **Country** |
| --- | --- | --- | --- | --- | --- | --- | --- |
| **Ciftci et al. 2018** | - ICDAS II - DIAGNOdent | **T0**: lesions with ICDAS code 2  **RI**: T0: 1.55+0.53 T3: 0.53+0.58 Difference T0-T3 (SD) (RevMan): 1.02 (1.52)  **FV** T0:1.85+0.36 T3: 1.57+0.59 Difference T0-T3 (SD) (RevMan): 0.28 (0.60) | - | **RI**: T0: 11.02+2.63 T3: 3.22+1.32  Difference T0-T3 (SD) (RevMan): 7.8 (11.62)  **FV** T0:12.25+2.73 T3: 6.0+2.42 Difference T0-T3 (SD) (RevMan): 1.02 (1.52) | - | **T0**:  no difference between RI and FV  **T0-T3**: RI significant decrease after infiltration for DIAGNOdent and ICDAS FV significant decrease after T3 for DIAGNOdent and ICDAS  **T3:** infiltration was more efficient compared with fluoride varnish according to DIAGNOdent pen and ICDAS scores" | Europe,  Turkey, Antalya |
| **Cronan et al. 2012** | -Modified enamel decalcification index (EDI) | **RI**: T6w: 24% of the lesion were totally masked Difference T0-T6w: 45,57  **NC** T6w:  Difference T0-T3: 6.25 OR (95%CI):2.38 (1.89-3.00) only within 6 month (OR2.21 (1.62-3.02)  **RI**: Improvement: Mean 0.5 (0.61) **NC**: Improvement: Mean 0.02 (0.32) | - | - | - | **T0**:  no difference between RI and NC  **T6w**:  Icon treatment decreased the size of many post-orthodontic white spot lesions compared to untreated controls | America, USA, Birmingham |
| **Gencer & Kırzıoglu 2019** | - spectrometry (Vista Easyshade) (ΔE) - tooth surface index of fluorosis (TSIF)  only for baseline evaluation) | - | **RI** T0: n/a T1 (or T0-T1?): 13.14 ± 0.76 T6 (or T0-T6?): 9.51 ± 0.80  **MA+CPP-ACP** T0: n/a T1 (or T0-T1?):7.92 ± 0.80 T6 (or T0-T6?):8.28 ± 0.84  **MA+TCP** T0: n/a T1 (or T0-T1?): 8.00 ± 0.84 T6 (or T0-T6?): 7.19 ± 0.88  It remains unclear if ΔE is calculated between two surfaces of one tooth or if it the change between two time points. presumably between time points (page 7) | - | - | **T6**: no significant difference between the groups | Europe, Turkey, Isparta |
| **First author** | **Measure of outcome** | **outcome: visual-tactile assessment** | **outcome: colorimetric analysis between the treated and sound enamel** | **outcome: DIAGNOdent** | **outcome:  demineralized area reduction** | **narrative results** | **Country** |
| Giray et al. 2018 | - DIAGNOdent | - | - | **RI**: T0: 12.96+4.22 T6: 5.96+3.38 Difference T0-T6: 7.0+3.67  **FV** T0:10.86+5.49 T6: 8.5+5.07 Difference T0-T6: 2.36+4.67 | - | **T0**:  no difference between RI and FV **T0-T6**: RI: Significant decrease in DIAGNOdent values between T0-T1d and T0-T6 FV: significant decrease in DIAGNOdent values between T0-T6 **T6**: Ri significant lower than FV | Europe,  Turkey, Istanbul |
| **Gözetici et al. 2018** | - LAA-ICDAS  - DIAGNOdent | **T0**: all lesion >7 **RI**: T0:100% active T6: 27.8% active Difference T0-T6 (SD) (RevMan): 27.8% (0.15)   **FV**: T0: 100% T6: 18.2% Difference T0-T6 (SD) (RevMan): 18.2% (0.20)   **P11-4**: T0: 100% T6: 41.2 Difference T0-T6:  **NC**: T0: 100% T6: 56.2  Difference T0-T6: | - | **RI**: T0: 33.2+17.33 T6: 9.95+9.11 Difference T0-T6: 23.25+18.21  **FV**: T0: 21.5+11.38 T6: 11.4+9.18 Difference T0-T6: 10.1+10.31   **P11-4**: T0: 24.4+13.55 T6: 16.26.14  Difference T0-T6: 8.15+13.89  **NC**: T0: 17.95+6.26 T6: 13.8+10.65  Difference T0-T6: 4.15+9.72 | - | **T0:** significant difference in baseline values  **T0-T6:** significant decrease in all groups decrease in RI was significantly higher than in other groups. Furthermore, decrease in FV was significantly higher than NC  **T6**: no significant difference between groups | Europe,  Turkey, Istanbul |
| **Gu et al. 2019** | - Spectroscopy (Crystaleye spectrophotometer) (ΔE) - Area ratio (R value, ratio of WSLs to the labial surface) | - | **RI** T0: 6.57 ± 2.48 T12: 2.20 ± 0.82  **MA** T0: 5.62 ± 2.04 T12: 2.26 ± 0.93 | - | R Value (Percentage (SD)): **RI**  T0: 36.13%+9.94% T12: 4.32%+3.69%  **MA** T0: 34.03%+13.66% T12: 12.82%+10.65% | **T0**:  no difference between RI and MA  **T12**:  The R value of resin infiltration was lower when compared with microabrasion at every recall point The DE had no significant differences between the two groups at any timepoint. | Asia, China, Chengdu |

| **First author** | **Measure of outcome** | **outcome: visual-tactile assessment** | **outcome: colorimetric analysis between the treated and sound enamel** | **outcome: DIAGNOdent** | **outcome:  demineralized area reduction** | **narrative results** | **Country** |
| --- | --- | --- | --- | --- | --- | --- | --- |
| **Gugnani et al. 2017** | - visual analog scale (VAS) (chance in esthetics (CE) and improvement in stains/opacities (IS)) | **RI** T0-T1d: CE: 5.50 ± 1.00 T0-T1d: IS: 4.98 ± 0.98  **RI (prolonged application time)** T0-T1d: CE: 5.53 ± 1.97 T0-T1d: IS: 5.18 ± 1.29  **Bleaching + RI** T0-T1d: CE: 5.35 ± 1.21 T0-T1d: IS: 4.40 ± 1.59  **bleaching** T0-T1d: CE: 1.90 ± 0.95 ** vs RI,  **RI(prolonged) and Bleaching+RI** T0-T1d: IS: 1.53 ± 1.07 ** vs RI, RI(prolonged) and Bleaching+RI ** p<0.001 | - | - | - | **T0**:  no significant difference between the groups  **T0-T1d**: all groups with resin infiltration were significantly better than without resin infiltration | Asia, India, Northern India |
| **Kannan & Padmanabhan 2019** | - DIAGNOdent - spectrometry (Vista Easyshade) (ΔE) |  | **Baseline**: n/a **RI**: T0-T6: 9.66 ± 1.42  **FV** T0-T6: 10.59 ± 1.21  ΔE is calculated between different time points, alteration of the teeth over time was presumably ignored | **Baseline**: 2-9 **RI**: T0: 4.48+1.42 T6: 1.48+0.81 Difference T0-T6 (SD) (RevMan): 3.0 (15.53)  **FV** T0: 4.6+1.29 T6: 1.51+0.72 Difference T0-T6 (SD) (RevMan): 3.09 (15.0) | - | **T6:**  No significant difference between RI an VF (Spectrometry)  **T0-T6:** RI: significant difference between 0 and 6 months  FV: significant difference between 0 and 6 months | Asia, India, Chennai |
| **Knösel et al. 2013,2018,2019** | - Spectroscopy (ShadePilot) (ΔE) | - | At 6 months: **RI**: T0: ΔE (SD) 7.88 (4.44) T6: ΔE (SD) 5.65 (2.83) Difference T0-T6 (SD): 2.55 (4.42)   **NC**: T0: ΔE (SD) 7.38 (4.34) T6: ΔE (SD) 7.6 (4.51) Difference T0-T6 (SD): 0.29 (2.38)   **RI-NC** T0: Mean(95%CI) 0.49 (-1-1.99) p=0.51 T6: Mean(95%CI) -1.953 (-3.34- -0.56) p=0.01  **At 24 months: RI:** T0: ΔE (SD) 8.76 (5.33) T24: ΔE (SD) 5.57 (2.6) | - | - | **T0**:  no significant difference between RI and NC **T0-T4**:  RI: significant difference between 0 and 6 months NC: no significant difference between 0 and 6 months Comparisons of T6, T12, and T24 with T0 yielded highly significant differences, whereas T6–T24 and T12–T24 differences were found to be not significant. | Europe Germany, Göttingen |

| **First author** | **Measure of outcome** | **outcome: visual-tactile assessment** | **outcome: colorimetric analysis between the treated and sound enamel** | **outcome: DIAGNOdent** | **outcome:  demineralized area reduction** | **narrative results** | **Country** |
| --- | --- | --- | --- | --- | --- | --- | --- |
| **Schoppmeier et al. 2018** | - colorimetric analysis (ΔE)  - patient satisfaction analog scale (VAS) - Thylstrup and Fejerskov index (TFI)  (only for baseline evaluation) | Patient satisfaction (Mean (SE)) **Bl+RI** T0-T1d: 1.77 (0.44) T0-T6:6.35 (0.18)  **NBl+RI** T0-T1d: 0.73 (0.44) T0-T6:5.78 (0.18)  **Difference BI+RI - NBI+RI** T6: -0.57 (0.26) correlation VAS - ΔE r=-0.42 | **Bl+RI** T0-T1d: -0.5 (0.77) T0-T6:-8.69 (0.14)  **NBl+RI** T0-T1d: 0.42 (0.77) T0-T6: -5.84 (0.14)  **Difference BI+RI - NBI+RI** T6: 2.85 (0.20) correlation VAS - ΔE r=-0.42 | - | - | **T0**:  no significant difference between Bl-RI and NBl-RI  **T6**: Bl-RI significantly reduced ΔE compared to NBl-RI,  Bl-RI non-significantly reduced VAS compared to NBl-RI | Europe, Germany, Cologne |
| **Senestraro et al. 2013** | -visual analog scale (VAS) of photographs - WSL area calculation. - ICDAS (results are not presented) | **T0**: lesions with ICDAS code 2 (n=62) or 3 (n=4)   **RI**: T0:(0+0) T8w: 65.9+26.6  **NC**: T0:(0+0) T8w: (SD) 9.2+15,8 | - | - | **RI**: T0: (0+0) T8w: (SD) 60.9% (24.2%)  **NC**: T0: (0+0) T8w: (SD) 1.0%+11,5% | **T0**:  Teeth that underwent treatment had significantly higher VAS ratings than did control teeth | America, USA, Portland |
