# Supplementary material for: Efficacy of resin infiltration to mask post-orthodontic or non-post-orthodontic white spot lesions or fluorosis — a systematic review and meta-analysis
Source: Clin Oral Investig. 2021 Jun 9;25(8):4711–9. doi: 10.1007/s00784-021-03931-7 (PMC8342329; doi:10.1007/s00784-021-03931-7)
Supplement: Supplementary file 3 — (PPTX 53 kb) [file 784_2021_3931_MOESM3_ESM.pptx]

## Slide 1
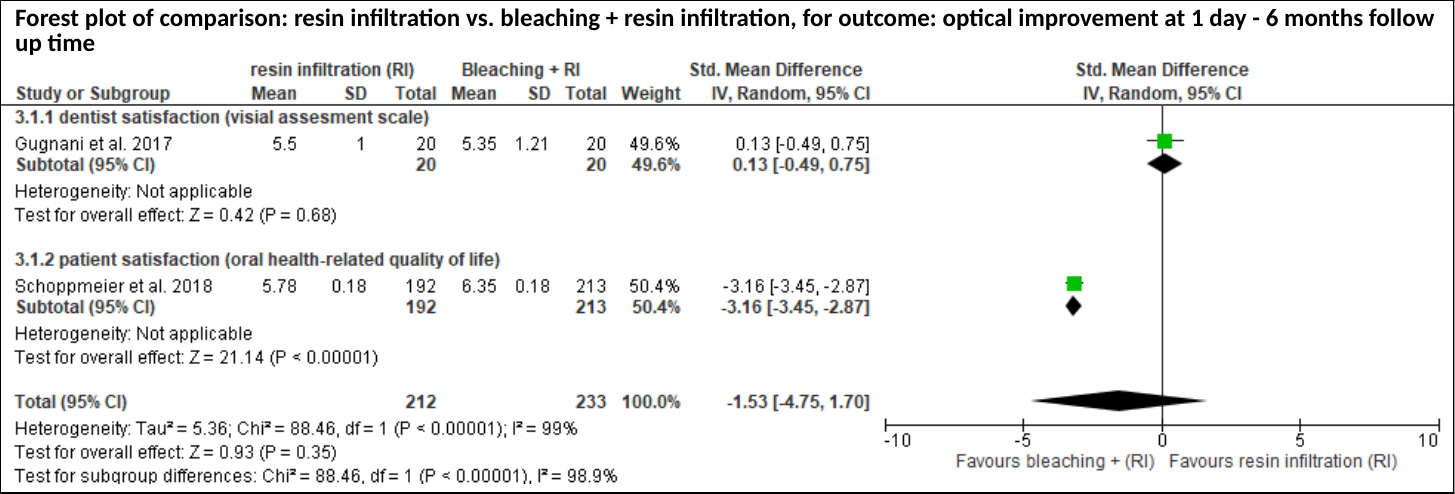

| Forest plot of comparison: resin infiltration vs. bleaching + resin infiltration, for outcome: optical improvement at 1 day - 6 months follow up time |
| --- |
